# NRF2 Regulation by Noncoding RNAs in Cancers: The Present Knowledge and the Way Forward

**DOI:** 10.3390/cancers12123621

**Published:** 2020-12-03

**Authors:** Federico Pio Fabrizio, Angelo Sparaneo, Lucia Anna Muscarella

**Affiliations:** Laboratory of Oncology, Fondazione IRCCS Casa Sollievo della Sofferenza, San Giovanni Rotondo, 71013 Foggia, Italy; a.sparaneo@operapadrepio.it (A.S.); l.muscarella@operapadrepio.it (L.A.M.)

**Keywords:** oxidative stress, NRF2, lncRNAs, miRNAs, tumor cells

## Abstract

**Simple Summary:**

The NRF2 pathway represents one of the most intriguing pathways that promotes chemo- and radioresistance of neoplastic cells. Increasing findings suggest that the NRF2 signaling can be modulated by multiple epigenetic factors such as noncoding RNAs, which influence a large number of oncogenic mechanisms, both at transcriptional and at post-transcriptional levels. As a consequence, the identification and characterization of specific noncoding RNAs as biomarkers related to oxidative stress may help to clarify the relationship between them and NRF2 signaling in the tumor context, in terms of positive and negative modulation, also referring to their intersection with other NRF2 crosstalking pathways. In this review, we summarize the recent updates on NRF2 network regulation by noncoding RNAs in tumors, thus paving the way toward the potential translational role of these small RNAs as key tumor biomarkers of neoplastic processes.

**Abstract:**

Nuclear factor erythroid 2-related factor 2 (NRF2) is the key transcription factor triggered by oxidative stress that moves in cells of the antioxidant response element (ARE)-antioxidant gene network against reactive oxygen species (ROS) cellular damage. In tumors, the NRF2 pathway represents one of the most intriguing pathways that promotes chemo- and radioresistance of neoplastic cells and its activity is regulated by genetic and epigenetic mechanisms; some of these being poorly investigated in cancer. The noncoding RNA (ncRNA) network is governed by microRNAs (miRNAs) and long noncoding RNAs (lncRNAs) and modulates a variety of cellular mechanisms linked to cancer onset and progression, both at transcriptional and post-transcriptional levels. In recent years, the scientific findings about the effects of ncRNA landscape variations on NRF2 machines are rapidly increasing and need to be continuously updated. Here, we review the latest knowledge about the link between NRF2 and ncRNA networks in cancer, thus focusing on their potential translational significance as key tumor biomarkers.

## 1. Introduction

The NRF2 protein, encoded by the Nuclear factor erythroid 2-related factor 2 (*NFE2L2*) gene, is a transcription factor endogenously expressed in eukaryotic cells and represents the main protector of the cellular antioxidant and cytoprotective response to harmful insults, as well as to xenobiotic damage and oxidative stress [1].

The activity of NRF2 is strictly dependent on a battery of transcriptional modulators that govern its physiologic activity under basal conditions as well as under internal and external stimuli arising from an oxidative stress condition [2]. In normal state, NRF2 orchestrates the maintenance of basal expression levels of more than 200 target antioxidant response element (ARE) genes through its direct link to this specific consensus sequences located at their promoter regions [3]. During the cellular adaptation to environmental modifications, the activation of NRF2 signaling is triggered by competitive interactions with the three ubiquitin ligase complexes—Cullin 3-RING-box protein-Kelch-like ECH-Associated Protein 1 (CUL3-RBX1-KEAP1), Skp1-cullin-F-box protein-transducin repeat-containing proteins (SCF/β-TrCP) and ERAD-associated E3 ubiquitin-protein ligase (HRD1). This complex interaction controls the ubiquitination and proteasomal degradation of NRF2 and, as a consequence, its abundance in specific subcellular compartments [4]. When NRF2 moves into the nucleus, it specifically binds to the ARE gene regions by heterodimerizing with small MAF (sMAF, avian musculo aponeurotic fibrosarcoma oncogene homolog) proteins and turning on the transcription of a large number of antioxidant and detoxification genes [5,6].

The main well-characterized mechanism of NRF2 regulation is tightly linked to its negative repressor, the KEAP1 protein. The KEAP1 is able to form an ubiquitin ligase complex with CUL3 and RBX1 to bind the NRF2 and enhancing its proteolitic degradation (in normal cell conditions) or detach from NRF2, because of KEAP1 cysteine modification (upon stress exposure) and favoring its nuclear translocation [7,8,9]. A KEAP1-independent modulation of the NRF2 signaling pathway may also occur both at transcriptional, post-transcriptional and at post-translational levels, with a consequent modification of its cellular localization, protein folding/stability and its DNA-binding ability [10,11].

Over the recent decades, the NRF2 transcription factor has been found to be overexpressed in various human disease, and a growing number of studies have identified abnormal NRF2 functions that go over the physiologic stress-regulating processes, including cancerous processes [12]. Fascinating scientific evidence indicates that the potential double role of NRF2 in cancer suppression and promotion [12,13] is under the control of genetic and epigenetic events, the latest ones strictly related to the activity of ncRNAs, such as microRNAs (miRNAs) and long noncoding RNAs (lncRNAs) [14,15]. These small pieces of RNAs are noncoding nucleotides that modulate transcriptional and post-transcriptional variations of oncogene and tumor suppressor genes, also not in physiologic processes such as cell differentiation, proliferation, cell cycle regulation, self-renewal capacity of stem cells and apoptosis, but also in the chemo- and radioresistance ability of tumors [16,17].

In the present review, we summarized the mechanisms by which ncRNAs affect NRF2 activity, their relationship with NRF2 regulators, their effects on ARE gene expression and other protein of different signaling pathways related to NRF2 activity.

## 2. Brief Overview of the NRF2 Signaling Pathway: Negative and Positive Regulators and ARE Genes’ Targets

The NRF2 transcription factor was firstly described thanks to its ability to bind to the NF-E2 site in the β-globin gene cluster and belongs to the cap’n’collar (CNC) subfamily of the basic region-leucine zipper (bZip) transcription factors (TFs) [18]. NRF2 protein counts seven NRF2-ECH homology domains, namely Neh1-Neh7, that are a highly conserved regions having different functions such as resistance to oxidative stress, autophagy, inflammation and inflammasome signaling modulation, apoptosis, mitochondrial biogenesis and stem cell maintenance [19].

The well-characterized mechanism that contributes in the regulation of NRF2 protein level is tightly linked to its negative repressor, Kelch-like ECH-associated protein 1 (KEAP1), which is able to modulate the cytoplasmatic levels of NRF2 by stimulating its proteolytic demolition or inhibiting this process thanks to the modification of NRF2 cysteine residues depending on cell status [7,8,20]. In normal cell conditions, NRF2 is kept in the cytosol by binding a dimer of KEAP1 protein, the main cysteine-rich protein that acts as a key sensor for oxidative/electrophilic stress molecules [21,22]. The KEAP1 guides NRF2 into the E3 ligase complex by means of bric-a-brac (BTB) and Kelch domains, which physically interact with CUL3 and NRF2. After the binding between NRF2 and E3 complexes, a polyubiquitination occurs at seven NRF2 lysine residues located in the Neh2 domain, followed by an ubiquitin-dependent degradation by the 26S proteasome [22,23]. Upon stimuli induction, the inactivation of KEAP1 occurs. As a result of this, the NRF2 translocates into the nucleus and dimerizes with sMAF proteins to bind the promoter region of several target antioxidants genes through their ARE consensus sequences [24].

In addition to the above-described master KEAP1 modulation, it has also been demonstrated that several complexes such as SCF/β-TrCP (S-phase kinase-associated protein 1 (SKP1), Cullin-1 (CUL1) and F-box protein E3 ubiquitin ligase are fundamental for the NRF2 stabilization process [25]. It has also been proved that p21 (CDKN1A), a well-known p53-downstream gene, can directly bind to ETGE and DLG motifs of NRF2, thus avoiding the interaction with KEAP1 and facilitating the NRF2 ubiquitination process [11]. Interestingly, the cyclin kinase inhibitor p21 and NRF2 are linked through a feedback mechanism, thanks to which it has been shown that the increase in p21 might increase the NRF2 activity [26]. Breast cancer type 1 susceptibility (BRCA1) protein also directly interacts with NRF2 and their binding impairs KEAP1-mediated NRF2 ubiquitination process, which is essential for NRF2’s stability and activation [27]. Lastly, in-depth investigations showed that the p62, also known as sequestosome 1 (SQSTM1), can mediate a selective autophagic KEAP1 degradation. These events trigger NRF2 through a positive feedback loop via p62/NRF2/KEAP1 pathway interactions, thus stimulating the transcription of several NRF2 ARE target genes [28,29].

## 3. NRF2 Deregulation in Cancer: Focus on the Epigenetic Modifications

Cancer development and progression depends on multiple genetic and epigenetic alterations. Specifically, the epigenetic processes are mechanisms that alter the expression level of the genes without modifying the sequence of DNA nucleotides. They include several changes in DNA methylation patterns, histone modifications and small noncoding microRNAs (miRNAs) and long noncoding RNAs (lncRNAs) expression [30].

A large amount of scientific evidence suggested that NRF2 favors the survival of normal cells as well as of cancer cells, thus corroborating the idea that its activation might promote the neoplastic progression. The identification of a “dark side of NRF2” has been debated over the time, but it still appears quite uncertain, since NRF2 could act both as a tumor suppressor and oncogene [12,13]. As for many genes, the occurrence of genetic and epigenetic modifications affecting the NRF2 pathway promotes cancer-related molecular events such as tumor initiation, growth, invasion and metastasis. The main interesting effect of all reported genetic/epigenetic alterations of the NRF2 pathway is a potential translational impact in terms of patients’ survival and response to chemo-radio and targeted therapies, firstly reported in lung tumors [31,32,33,34,35]. The biallelic inactivation of the *KEAP1* gene was reported to have a great impact on the upregulation of NRF2 and was firstly described in non small cell lung cancer (NSCLC) and then widely notified in many other solid tumors [36,37,38,39]. Point missense mutations of *NFE2L2* gene were also reported to have a similar effect on the KEAP1/NRF2 binding affinity and frequently recurred not only in lung cancer [40], but also in head and neck carcinoma [41], hepatocellular carcinoma (HCC) [42], papillary renal cell carcinoma (PRCC) [43] as well as esophageal and skin cancers [44]. In lung squamous carcinoma (LUSC) and head and neck cancers, an alternative splice variant of *NFE2L2* gene lacking exon 2 was described to play a significant role in the loss of interaction of NRF2 with the Kelch domain of *KEAP1*. This in turn caused the stabilization of NRF2 and the induction of its transcriptional response [45]. Epigenetic modifications have been widely described to impact on the modulation of the KEAP1/NRF2 system in cancer. Aberrant methylation at the promoter island of the *KEAP1* gene has been widely reported as one of the most important mechanisms of *KEAP1* silencing in solid tumors, such as glioma, breast, prostate, colorectal, thyroid cancers, clear renal cell carcinoma and lung cancer and was linked to tumor development, chemoresistance and mortality risk [14]. More recently, in NSCLC Kirsten rat sarcoma viral oncogene homolog (*KRAS*) wild-type subpopulations, a novel and significant correlation was observed among promoter and in intragenic exon 3 cytosine-guanine dinucleotide island (CpG-I) methylation and the transcription levels of *KEAP1, NFE2L2* and many ARE genes [46,47].

Given the real complexity and multiplicity of the cellular processes controlled by NRF2, one of the most recent and attractive pieces of evidence suggests that the regulation NRF2 can be also guided by the ncRNAs, such as miRNAs and lncRNAs [48]. The following two main sections will summarize the findings about ncRNAs related to oxidative stress and their relationship with the NRF2 network.

## 4. LncRNAs Intercepting the NRF2 Axis

LncRNAs are noncoding RNA molecules not more than 200 nucleotides in length involved in the inhibition of gene expression as well as in the regulation of many different physiological mechanisms such as cell differentiation, cycle regulation, apoptosis and proliferation [49,50].

lncRNAs are characterized by higher stage and cell subtype specificities in various tumors. Since lncRNAs participate in several cells signaling pathways and lead to the alteration of tissue-specific pattern of expression, their importance is related to the development of novel strategies for specific cancer subtype diagnosis and targeting. The modulation of a large number of lncRNAs, which could be up- or downregulated in several cancers, is important to determine their tissue specificity and for linking them to tumor stage [51].

**Figure 1:**
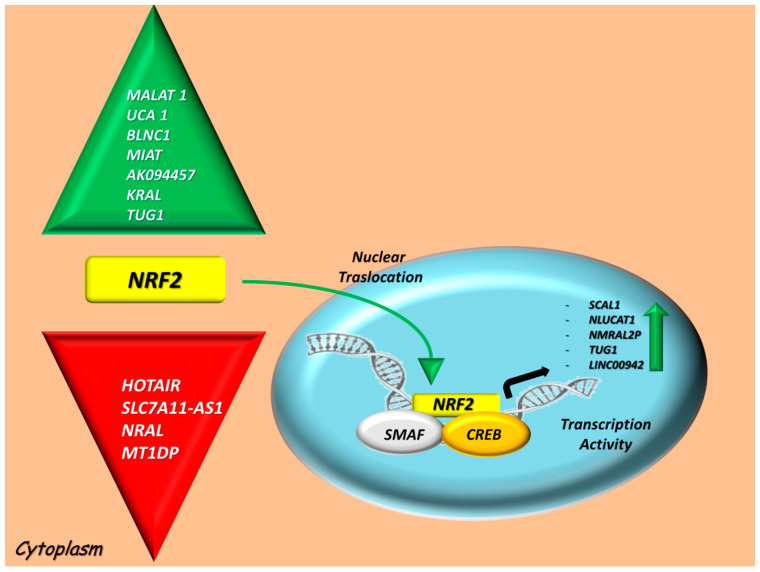
Experimentally validated long noncoding RNAs (lncRNAs) which contribute to a down and upregulation of Nuclear factor erythroid 2-related factor (NRF2) at mRNA/protein levels in cancers, based on their regulatory (left side) or transcriptional activities (right side). Looking at the regulatory lncRNA list, some of them induce an overexpression of NRF2 (Metastasis-associated lung adenocarcinoma transcript (MALAT1), Urothelial carcinoma-associated 1 (UCA1), BLNC1, Myocardial infarction-associated transcript 1 (MIAT), AK094457, KEAP1 regulation-associated lncRNA (KRAL), Taurine-upregulated gene 1 (TUG1); green arrow), whereas another group causes a reduction in NRF2 levels (Hox transcript antisense intergenic RNA (HOTAIR), Solute Carrier Family 7 Member 11-antisense 1 (SLC7A11-AS1), NRF2 regulation-associated lncRNA (NRAL), metallothionein 1D pseudogene (MT1DP); red arrow). On the other hand, NRF2 stimulates the transcription of the following lncRNAs: SCAL1, nuclear smoke- and cancer-associated lncRNA (NLUCAT1), NmrA-like redox sensor 2 pseudogene (NMRAL2P), TUG1 and long intergenic nonprotein coding RNA 942 (LINC00942). Abbreviations: sMaf, avian musculo aponeurotic fibrosarcoma oncogene homolog; CREB, cAMP response element-binding protein; NRF2, Nuclear factor erythroid 2-related factor 2; HOTAIR, Hox transcript antisense intergenic RNA; MALAT1, Metastasis-associated lung adenocarcinoma transcript 1; UCA1, Urothelial carcinoma-associated 1; MIAT, Myocardial infarction-associated transcript 1; SLC7A11-AS1, Solute Carrier Family 7 Member 11-antisense 1; KRAL, KEAP1 regulation-associated lncRNA; NRAL, NRF2 regulation-associated lncRNA; TUG1, Taurine-upregulated gene 1; MT1DP, metallothionein 1D pseudogene; SCAL1 or LUCAT1, smoke- and cancer-associated lncRNA 1; NLUCAT1, nuclear smoke- and cancer-associated lncRNA 1; NMRAL2P, NmrA-like redox sensor 2 pseudogene; LINC00942, Long Intergenic Nonprotein Coding RNA 942.

LncRNAs may also play a pivotal role in the onset, progression and stem cell pluripotency of cancer cells [52] and in the oxidation/antioxidant system by acting as negative or positive regulators via NRF2 interactions [53]. According to these, we identified two different groups of lncRNAs linked to the NRF2-oxidative stress system: transcriptional targets and regulators that are summarized in Figure 1.

### 4.1. LncRNAs Modulated by NRF2 Signaling

Emerging reports on NRF2-associated lcRNAs described a large number of identified transcriptional targets of NRF2, but only a small part of these were functionally validated. The most well-characterized LncRNAs whose transcription were directly modulated by NRF2 include SCAL1 (LUCAT1), nuclear smoke- and cancer-associated lncRNA (NLUCAT1), NmrA-like redox sensor 2 pseudogene (NMRAL2P), Taurine-upregulated gene 1 (TUG1), and long intergenic nonprotein coding RNA 942 (LINC00942) and are listed in Table 1.

#### 4.1.1. SCAL1 (LUCAT1)

The smoke- and cancer-associated lncRNA 1 (*SCAL1* or *LUCAT1*) is a cancer lncRNA related to smoke exposure that is strictly associated with NRF2 activity. The NRF2 binds the *SCAL1* promoter region through a putative nuclear factor-erythroid-2 (NFE2) binding site, thus suggesting that this lncRNA could be transcriptionally regulated by NRF2. On the other hand, the silencing of *SCAL1* in human bronchial epithelial cells (HBE) induces a significant enhancement of cytotoxicity under the cigarette smoke extract (CSE) exposure. These findings suggested that *SCAL1* may be considered as a downstream mediator of NRF2 against reactive oxygen species (ROS)-induced oxidative stress in airway epithelial cells [54]. Additionally, a transcriptomic analysis in a large cohort of adenocarcinoma and squamous cell carcinoma tumors of the lung showed that *SCAL1* correlated with *KEAP1* and *NFE2L2* mutational statuses [55].

#### 4.1.2. NLUCAT1

The *NLUCAT1* is a nuclear transcript variant of *LUCAT1* that is composed of six exons, which are under the control of NRF2 transcription factors. In the A549 lung adenocarcinoma (LUAD) cell line, it was observed that the targeted deletion of *NLUCAT1* led to a decrease in cell proliferation and invasion, increasing the oxidative stress and cisplatin-induced NRF2-mediated apoptosis. More interestingly, a recent transcriptomic analysis revealed that *NLUCAT1* may exert a positive loop on the NRF2 network [56].

#### 4.1.3. NMRAL2P

NmrA-like redox sensor 2 pseudogene (*NMRAL2P*) is the first functional pseudogene that was identified as a direct target of NRF2 and downstream regulator of NRF2-dependent NQO1 activation in sulforaphane (SFN)-treated colon cancer cells. By using functional and pharmacological assays, it was observed that siRNA-mediated knockdown of *NFE2L2* gene was able to interfere with the ability of SFN to induce *NMRAL2P* expression [57].

#### 4.1.4. TUG1

Taurine-upregulated gene 1 (*TUG1*) has been reported to be involved in different pathogenic cellular mechanisms, as well as carcinogenesis and chemoresistance of cancer cells [58]. In prostate and esophageal squamous cell carcinoma (ESCC), *TUG1* directly binds NRF2, thus upregulating its expression at protein level together with its downstream members (HO-1 and NQO1) [76]. The silencing of *TUG1* could offer an interesting point of view aimed at overcoming drug resistance in ESCC using NRF2 inhibitors [73]. Moreover, new recent evidence also suggested a synergic and oncogenic role of NRF2 and TUG1 in stimulating proliferation, apoptosis, migration and resistance to doxorubicin treatment in urothelial carcinoma of the bladder (UCB) cells [59].

#### 4.1.5. LINC00942

Little evidence is now available on the role of long intergenic nonprotein coding RNA 942 (*LINC00942*) in the modulation of the NRF2 antioxidant pathway. Transcriptomic analyses demonstrated that *LINC00942* could directly interact with NRF2 through its binding to the *NFE2L2* promoter region, thus decreasing the Glutamate-Cysteine Ligase Catalytic Subunit (GCLC) mRNA and protein levels. This observation has been linked to the fact that human tumors having *NFE2L2* gain-of-function mutations showed an increased expression of *LINC00942* [60].

### 4.2. Positive and Negative LncRNA Regulators of the NRF2 Activity

This group of LncRNA participates in the recruitment of chromatin modifiers [77] or transcription factors [16] and can modulate post-transcriptional events, as well as the translational repression and/or the enhance mRNA degradation, or serve as natural “miRNA sponges” through sequestration of miRNAs [78,79]. They are also involved in the modulation of the oxidation/antioxidant system by acting as negative or positive regulators via NRF2 interactions, as explained below and summarized in Table 1.

#### 4.2.1. HOTAIR

The first evidence about the regulation of NRF2 expression by Hox transcript antisense intergenic RNA *(HOTAIR*) was provided by Zhang and coworkers, who demonstrated that *HOTAIR* regulates NRF2 levels via mediating histone H4 acetylation at the *NFE2L2* gene promoter in Gc1-Spg (germ cell-1 spermatogonial) cells. Once the silencing of *HOTAIR* occurred, a positive correlation between the downregulation of NRF2 levels and H4 acetylation within the *NFE2L2* promoter was observed [61]. In addition, it is hypothesized that *HOTAIR* participates in the epigenetic suppression of the NRF2-multidrug resistance-associated protein 2/4 (MRP2/4) pathway and stimulates the transcription of a lot of ARE genes [62].

#### 4.2.2. MALAT1

A cooperation between the KEAP1/NRF2 axis and the lncRNA metastasis-associated lung adenocarcinoma transcript 1 (*MALAT1*) was recently documented in human umbilical vein endothelial cells (HUVECs). Under hydrogen peroxide exposure, the expression of MALAT1 stimulates the downregulation of KEAP1 at mRNA and protein levels, thus leading the NRF2 nuclear accumulation and ARE target gene activation [63]. *MALAT1* can also directly bind NRF2, thus activating the NRF2 signaling cascade and ARE-related gene upregulation [64]. Moreover, *MALAT1* also interacts with Polycomb repressive complex 2 (PRC2) components, including Enhancer of zeste homolog 2 (EZH2). In fact, Li and colleagues showed in lung cancer cells that EZH2 targets *NFE2L2* promoter, thus suppressing its expression by regulating trimethylation of histone H3 at lysine 27 (H3K27me3) [80].

#### 4.2.3. UCA1

The Urothelial carcinoma-associated 1 (*UCA1*) lncRNA was associated with miR-495 activity, which targets and negatively regulates it in renal cell carcinoma, after the epigenetic repression of p21 [65]. Li and colleagues showed that a high expression of UCA1 could develops cisplatin resistance in lung cancer cells via UCA1/miR-495/NRF2 crosstalk [66]. In hippocampal tissues and neurons, UCA1 activates NRF2-dependent cytoprotective pathway by sponging miR-495 and, overall, leads to an inhibition of apoptosis during seizure-induced brain injury [67].

#### 4.2.4. BLNC1

LncRNA *BLNC1* was found overexpressed in diabetic nephropathy (DN) and contributes to attenuate chemical and physical insults, inflammation and renal fibrosis by hyperactivating the NRF2/HO-1 pathway in renal HK-2 proximal tubules kidney cells. Since the BLNC1 could also inhibit the activation of the nuclear factor kappa-light-chain-enhancer of activated B cells (NF-κB) pathway, it was suggested that the crosstalk between NRF2/HO-1 and NF-κB pathways might serve as a potential therapeutic target against DN [81].

#### 4.2.5. MIAT and AK094457

The lncRNA Myocardial infarction-associated transcript (*MIAT*) targets NRF2 by mediating the high glucose-induced tubular injury in renal HK-2 epithelial cells and modulates cell viability in proximal tubules in renal via balancing NRF2 levels [68].

The downregulation of *AK094457* was found to be inversely correlated to the upregulation of NRF2 and HO-1 in vascular smooth muscle cells (VSMCs) and this may suggest how both are involved in antioxidant mechanisms [69].

#### 4.2.6. SLC7A11-AS1

Recent evidence about the relationship between Solute Carrier Family 7 Member 11-antisense 1 (*SLC7A11-AS1*) and post-transcriptional regulation of NRF2 via protein–protein interactions was reported in pancreatic cancer. The *SLC7A11-AS1* was observed to hamper with the stability of NRF2, thus impairing the E3 Ub-ligases processes as well as targeting proteins for degradation and controlling protein turnover by modification of ubiquitin–proteasome system (UPS)-related proteins. In pancreatic ductal adenocarcinoma cells (PDACs), it was observed that the block of SCFβ-TRCP-mediated degradation of NRF2 produced the reduction in intracellular ROS and stimulated stemness and chemoresistance properties [70].

#### 4.2.7. KRAL and NRAL

The dual and opposite roles of NRF2 regulation-associated lncRNA (*NRAL*) and KEAP1 regulation-associated lncRNA (*KRAL*) have been recently outlined in hepatocellular carcinoma (HCC) cell lines. *KRAL* activation promotes chemo-sensitization to HCC cells by targeting the miR-141/KEAP1 axis as a competing endogenous RNA (ceRNA), with a consequent upregulation of *KEAP1* and a silencing of *NFE2L2*, respectively [71]. On the other hand, *NRAL* inhibits the miR-340-5p/NRF2 crosstalk by exerting a transcriptional repression through an interaction with the *NFE2L2* 3′-untranslated (3′UTR) regions and this causes an increase in its expression and drug resistance on HepG2 cells [71,72].

#### 4.2.8. MT1DP

An intriguing hypothesis about a potential correlation between metallothionein 1D pseudogene (*MT1DP*) lncRNA and NRF2 was provided by Gao and colleagues. The main finding was that *MT1DP* sustained the activation of miR-365 through a 3′UTR consensus site, which in turn, is able to repress the *NFE2L2* gene and impair cadmium-stimulated oxidative stress [74]. More recently, it was reported that MT1DP weakened the expression of NRF2 and enhanced the sensitivity of NRF2-overexpressed NSCLC cells to erastin-induced ferroptosis by stabilizing miR-365a-3p [75].

## 5. miRNAs Intercepting the NRF2 Axis

miRNAs are proximately 22 nucleotides single-stranded noncoding RNA molecules able to modulate the translation or stability of mRNA molecules through interaction with specific mRNAs with complementary base sequences [82]. In human cancer, the miRNA machinery is deregulated by several mechanisms and impacts on the tumorigenic activities of neoplastic cells mainly promoting cell invasion and metastasis [83].

**Figure 2:**
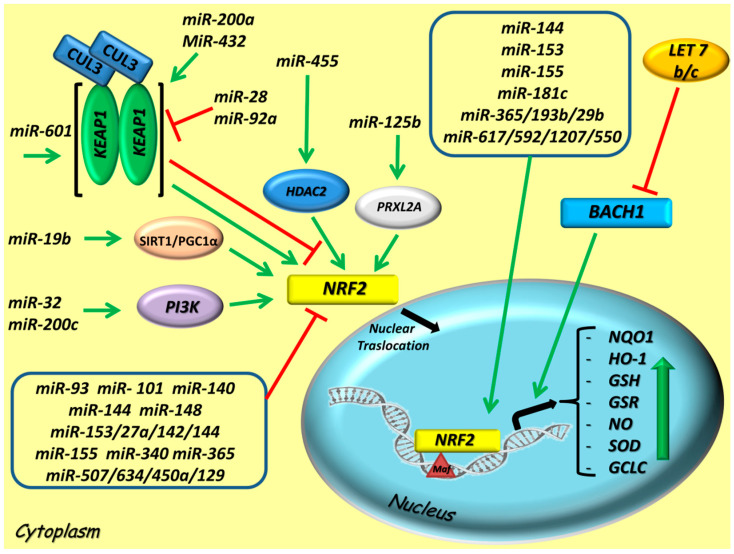
Modulation of the NRF2 signaling pathway by microRNAs (miRNAs) in cancers. Representative scheme depicts miRNAs that directly or indirectly target NRF2 pathway, thus impacting on the expression of antioxidant response element (ARE) genes. The red lines indicate an inhibitory effect on NRF2, otherwise the green lines a stimulatory effect on NRF2. A group of miRNAs with NRF2 indirect interaction is indicated in the bottom box, whereas those which stimulate NRF2 direct interaction in the upper box. Abbreviations: CUL3, Cullin 3; KEAP1, Kelch-Like ECH-Associated Protein 1; NRF2, Nuclear factor erythroid 2-related factor 2; sMAF, avian musculo aponeurotic fibrosarcoma oncogene homolog; SIRT1/PGC-1α, SIRT1, Sirtuin 1/PGC-1α, Peroxisome proliferator-activated receptor gamma coactivator 1-alpha; PI3K, Phosphoinositide 3-kinase; HDAC2, Histone deacetylase 2; PRXL2A, Peroxiredoxin-like 2A; BACH1, bric-a-brac (BTB) and cap’n’collar (CNC) homology, basic leucine zipper transcription factor; NQO1, NAD(P)H Quinone Dehydrogenase 1; HO-1, Heme oxygenase-1; GSH, glutathione; GSR, Glutathionereductase; NO, nitric oxide; SOD, superoxide dismutase; GCLC, Glutamate-Cysteine Ligase Catalytic Subunit.

The exact molecular mechanisms by which miRNAs can modulate the antioxidant defense system in cells are only partially understood. However, the most recent scientific evidence suggests that several miRNAs crosstalking with the NRF2 signaling pathway by directly regulating the expression of NRF2, by influencing the nuclear translocation of NRF2 or by indirectly modulating KEAP1 and other upstream mediators of the NRF2 pathway [84,85] (Figure 2).

### 5.1. Positive miRNA Regulators of NRF2 Activity

The first miRNAs reported to exert a positive stimulation on NRF2 transcription were let 7b and let 7c, which surely regulate the transcription factor BTB and CNC homology 1 (*BACH1*) expression in human hepatoma Huh-7 and liver cells. As a result, an increase of HO-1 expression mediated by NRF2 and an attenuation of the oxidative stress mechanism were demonstrated [86,87].

In LO2 liver cells, a reduction in miR-19b levels was observed under acetaminophen (APAP) treatment. This, in turn, leads to the stimulation of sirtuin-1 (*SIRT1*), which activates the NRF2 cascade and its related downstream target genes and enhances drug hepatotoxicity [88].

miR-32 contributes to the activation of Phosphoinositide 3-kinase (*PI3K*) in prostate cancer which, in turn, upregulates NRF2. In this way, NRF2 creates a positive feedback loop by promoting survival in cultured human retinal pigment epithelium (RPE) cells [89].

In oral squamous cell carcinoma (OSCC), it was observed that the overexpression of the Peroxiredoxin-like 2A (*PRXL2A*) gene was induced by miR-125b, which successively suppresses the oxidative stress damage driven by positive feedback loops involving the NRF2 signaling pathway [90].

In neuroblastoma SH-SY5Y cells, the transfection experiments with a miR-144 mimic was seen to accelerate apoptosis and reduced the expression of several enzymes regulated by NRF2 that are involved in glutathione (GSH) synthesis and reactive oxygen species scavenging [91].

Evidence of the link between miR-153-3p and NRF2 emerged in breast cancer cell lines, where lower miR-153-3p expression levels correlated to an increase in NRF2 and downstream gene levels with a consequent stimulation of tumor cell migration and invasion [92].

In lung cancer cell lines it has been found that miR-155 confers resistance to arsenic trioxide (ATO) by activating the NRF2 signaling pathway. The consequent upregulation of HO-1 and NQO1 inversely correlated with a relative decrease in apoptosis, thus promoting the enhancement of the B-cell lymphoma 2/BCL2-Associated X protein (BCL-2/BAX) ratio [93].

Bioinformatics predictions coupled with functional analysis revealed the presence of a miR-181c binding site in the 3′UTR of *NFE2L2*, thus enhancing the activation of multiple pro-survival pathways.

The family of miR-200 may play an important role in the KEAP1-dependent mechanism of NRF2 regulation. It has been demonstrated that miR-200a stimulates the NRF2 signaling pathway by suppressing *KEAP1*, mainly due to the decrease in ROS concentration, thus ensuring the survival of osteoblasts upon dexamethasone treatment in osteoblast (OB)-6 cells [94].

It is well-known that the Zinc Finger E-Box Binding Homeobox 1 (ZEB1) protein could inhibit E-cadherin, thus promoting the epithelial mesenchymal transition (EMT) and the overexpression of NRF2 [95]. miR-200c was also observed in both breast and ovarian cancers to induce AKT upregulation. Given that the upstream regulation of PI3K/AKT pathway activates NRF2 and its related antioxidant genes, the downregulation of miR-200c by NRF2 could potentially induce a negative feedback mechanism [89].

miR-365, miR-193b and miR-29b are also involved in the scenario of cancer NRF2-related deregulation. Enforced expression of miR-29b occurs in response to NRF2 activation [96,97]. The presence of a feedback loop between NRF2 and miR-29b was suggested by Chan and colleagues, who firstly described the localization of the Sp1 transcription factor binding site at the *NFE2L2* promoter region. NRF2 could suppress miR-29b and induce an upregulation of both SP-1 and NRF2, thus protecting cells from apoptosis [98,99].

Evidence of a positive modulation of NRF2 by miR-432-3p was reported in esophageal squamous cell carcinoma (ESCC). By using ARE reporter plasmids, miR-432-3p was supposed to promote the NRF2 protein stabilization by directly binding the *KEAP1* coding region, thus increasing the resistance of cancer cells to cisplatin (CDDP) [100].

miR-455-3p activates NRF2 via its upstream mediator, the histone deacetylase 2 (*HDAC2*), thus ensuring more protection to osteoblasts against oxidative injury by activating NQO1, HO-1 and GCLC expressions [101,102].

miR-601 targets *CUL3* and contributes to the activation of the NRF2 signaling pathway in protecting retinal pigment epithelium (PRE) cells against oxidative damage. The study of the effects of miR-601 expression on CUL3 and NRF2 signaling revealed that this miRNA could participate in a dependent CUL3-KEAP1-NRF2 activation mechanism, thus suggesting a possible therapeutic strategy for treatment or prevention of Age-related macular degeneration (AMD) [103].

Finally, the group containing miR-617, miR-592, miR-1207 and miR-550 was recently correlated to the NRF2 activity in different cancer profiles. Specifically, it was found that NRF2 upregulates miR-617, thus protecting the esophageal cancer cells from chemical and physical insults. It has also been observed that NRF2 is involved in a drug resistance mechanism in colorectal and liver carcinoma, thus suggesting a possible link between the reduction in miR-592 levels and NRF2 activation in these tumors [104,105]. In prostate cancer, a concomitant and synergic upregulation of miRNA-1207 and NRF2 was demonstrated [106].

Likewise, little is known about miR-550. The bioinformatics DIANA-microT V3.0 tool was used to identify downstream NRF2 targets, such as tumor suppressor BAF Chromatin Remodeling Complex Subunit BCL11B (*BCL11B*), which correlates to miR-550 activity, thus corroborating the importance of NRF2 upregulation and promoting cell growth and proliferation [107].

miRNA-positive regulators of NRF2 signaling are presented in Table 2.

### 5.2. Negative miRNA Regulators of NRF2 Activity

The first investigated NRF2-related miRNA in MCF-7 breast cancer cells was miR-28, which exerts a negative effect on NRF2, independently from its master regulator, the KEAP1 protein [109].

An inverse relationship was proposed for miR-92a expression, whose suppression induces the activation of the NRF2-KEAP1-ARE pathway in cultured human umbilical vein endothelial cells (HUVECs). By luciferase assays, it has been demonstrated that miR-92a was able to promote cell proliferation, thus contributing to a decrease in apoptosis-inducing factors such as caspase 3, tumor necrosis factor (TNF)-α and KEAP1 expression with an upregulation of NRF2 and many of its ARE target genes [110].

Singh and colleagues suggested that miR-93 could act as an oncogenic negative regulator of NRF2 activity by targeting specific sites at the 3′UTR region in MCF-10A breast cancer cells. To prove the tumor-enhancer effect of miR-93 mediated by NRF2 signaling, the authors proved an inverse correlation between an inhibition of miR-93 and an increased expression of NRF2 with a consequent significant reduction in mammosphere propagation, apoptosis and DNA damage upon 17β-estradiol treatment [111].

Interesting evidence also came from the study on miR-101 in breast cancer. The miR-101 binds to the 3′UTR region of *NFE2L2*, thus influencing its expression, promoting the suppression of cell proliferation and enhancing the sensitivity of cancer cells to ROS [112].

NRF2/miR-140 crosstalk may play an important role in major protection in fibrotic lung tissues against transforming growth factor (TGF)-β1-mediated inflammation and fibroblast differentiation induced by radio treatment [113,114].

The question looks intriguing when we looked at several studies focusing on miR-144. This miRNA was seen to be upregulated not only in the peripheral blood of acute myeloid leukemia (AML) affected patients but also in AML HL-60 cells, thus suggesting that its inhibition can promote apoptosis and suppress *NRF2* activation [115]. Of interest, the molecular link between NRF2 and miR-144 correlates to an enhancement of toxicity of 5-fluorouracil (5-FU) in hepatocellular cancer cell lines [116].

Qu and colleagues recently proposed a novel mechanism of NRF2 regulation in response to oxidative stress in endometrial cancer cells via miR-148b. They observed that HIF-1 and NRF2 expressions were significantly reduced upon miR-148b overexpression; endoplasmic reticulum metalloprotease 1 (*ERMP1*) related and suggested a tumor-suppressive role of this miRNA in RL95-2 cells of human endometrial cancer [117].

Another piece of evidence of a negative regulation of NRF2 by miRNAs was provided by Wang and colleagues, when they observed that miR-153 acts as an oncogenic effector of the apoptosis, thus reinforcing the proliferation ability of breast cancer cells via suppression of *NRF2* and its downstream ARE genes’ expression [92]. Finally, alternative approaches, such as in silico and in vitro models of cancer cell lines, added novel molecular insights and elucidated the link between NRF2 and miRNAs. A group of novel miRNAs (miR-153/miR-27a/miR-142-5p/miR-144) was identified as exerting a modulator effect on NRF2 expression and on oxidative stress balance in neuronal cells. The upregulation of this group of miRNAs resulted in suppressing *NRF2* signaling, thus turning off both glutamate-cysteine ligase catalytic (GCLC) and glutathione-disulfide reductase (GSR) expressions [118].

In bronchial epithelial cells, it was demonstrated that, under arsenic treatment, the activity of miR-155 directly accelerates apoptosis by blocking NRF2 activity and those of related cytoprotective genes as well as glutathione (GSH), nitric oxide (NO) and superoxide dismutase (SOD) [119]. Moreover, a notable correlation between the overexpression of miR-155 and the radiation-induced pulmonary fibrosis by NRF2 was reported in a mouse model [120].

In human neuroblastoma SH-SY5Y cells, the miR-181s are able to suppress the Sirtuin 1/Peroxisome proliferator-activated receptor gamma coactivator 1-alpha/NRF2 (*SIRT1/PGC-1α/NRF2*) signaling pathway [121], whereas in hepatocellular carcinoma patterns it was shown that miR-340 targets the NRF2 pathway and alters the chemoresistant phenotype of cells after cisplatin treatment [122].

The molecular bridge between metallothionein 1D pseudogene (*MT1DP*) and NRF2 was linked to the miR-365 activity in human hepatocellular carcinoma cell line HepG2. Upon cadmium-induced oxidative stress, the upregulation of miR-365 acts as a sensor for MT1DP activation, thus thereby repressing the NRF2 activity via direct binding to its 3′UTR [74].

Lastly, miR-507, miR-634, miR-450a and miR-129-5p were shown to negatively modulate to NRF2 oncogenic activity by directly targeting *NFE2L2* in the neuroblastoma SH-SY5Y cell line [123], whereas it was demonstrated that they could exert a synergic effect of increasing sensitivity to cisplatin treatment in NSCLC A549 cells [124].

As follows, Table 3 shows miRNA-positive regulators of NRF2 signaling.

## 6. Conclusions and Perspectives

It is becoming increasingly clear that the NRF2 signaling pathway can be epigenetically modulated in different ways and that the identification of new sensitive and reliable biomarkers (miRNAs and lncRNAs) that guide this mechanism in solid tumors could be useful to better understand the link between oxidative stress and cancer. This review aimed to provide a concise summary of the most recent updates in this field, but further massive investigations are required to allow the translational utility of ncRNAs as potential biomarkers related to NRF2 activity.

The oxidative stress processes related to the NRF2 activity have become a main issue in cancer biology investigation over recent years, so the identification of ncRNA alterations related to these dynamic processes will surely provide new opportunities to understand cancer biology and treatment. Moreover, further characterization of the fundamental mechanisms by which altered processing of these specific ncRNAs contributes to tumor onset and progression will be crucial to ensure great effects of many promising cancer therapies that specifically target the altered processing of RNA, with minimal effects on normal cells. From a practical and more futuristic point of view, the advent of next-generation sequencing (NGS) and liquid biopsy techniques able to detect point mutations and ncRNA level variations in blood will also allow a better clarification of the existing relationship between ncRNAs and the NRF2 pathway in a dynamic and longitudinal monitoring context of cancer patients during the course of the disease.

## Figures and Tables

**Table 1:** (**A**). LncRNAs modulated by NRF2 signaling. (**B**). Positive and negative lncRNA regulators of the NRF2 activity.

(**A**)						
**LncRNA ID**	Chr Location *	**Role**	**NRF2 Levels**	**NRF2-Related Effects**	**Cell Cultures/Validation Models**	**Refs.**
*SCAL1 (LUCAT1)*	chr5: 91,285,160–91,314,516	Transcriptional activity	Upregulated	Downstream mediator of NRF2 against ROS-induced oxidative stress, having a pro-tumorigenic role	HBE cells; Lung ADC and SqCC	[54,55]
*NLUCAT1*	5q14.3	Transcriptional activity	Upregulated	It induces a positive loop on the NRF2 network, correlates with hypoxic stress and contributes to tumor invasion and proliferation	A549 ADC cells	[56]
*NMRAL2P*	chr3: 185,959,943–185,980,872	Transcriptional activity	Upregulated	Downstream regulator of NRF2-dependent NQO1 activation. It exerts an oncogenic action by promoting EMT	Colon cancer cells	[57]
*TUG1*	chr22: 30,970,182–30,978,847	Transcriptional activity	Upregulated	Oncogenic roles in apoptosis, proliferation and chemoresistance by modulating the NRF2 signaling	UCB cells; prostate cancer cells	[58,59]
*LINC00942*	chr12: 1,500,525–1,504,424	Transcriptional activity	Upregulated	It concurs on NRF2 antioxidant pathway activation	19 cancer types from TCGA	[60]
(**B**)						
**LncRNA ID**	Chr Locations *	**Role**		**NRF2-Related Effects**	**Cell Cultures/Validation Models**	**Refs.**
*HOTAIR*	chr12: 53,962,308–53,974,956	Negative regulator		Mediates histone H4 acetylation at the *NFE2L2* gene promoter and is also involved in the epigenetic suppression via NRF2-MRP2/4 pathway	GC-1 spg; human hepatocytes L02 cells	[61,62]
*MALAT1*	chr11: 65,497,688–65,506,431	Positive regulator		Participates in MALAT1-mediated HUVEC protection from hydrogen peroxide and it is negatively regulated by KEAP1	HUVEC cells; mouse primary hepatocytes	[63,64]
*UCA1*	chr19: 15,828,206–15,836,136	Positive regulator		It has an oncogenic role promotes proliferation through p21 repression, it enhances chemoresistance to cisplatin via UCA1/NRF2 crosstalk signaling by sponging miR-495 and promoting the inhibition of apoptosis	Human RCC cells; A549 lung ADC cells; HEK-293 cells	[65,66,67]
*BLNC1*	chr9: 95,559,657–95,568,023	Positive regulator		Participates in the hyperactivation of HO-1/NRF2 and crosstalks with NF-κB pathway by attenuating renal fibrosis, inflammation and oxidative stress	Renal HK-2 cells	[62]
*MIAT*	chr22: 26,657,520–26,676,475	Positive regulator		Targets NRF2 by mediating high glucose- induced tubular injury	renal HK-2 epithelial cells	[68]
*AK094457*	chr10: 124,745,200–124,748,551	Positive regulator		Inversely correlates with the upregulation of NRF2 and HO-1	VSMC cells	[69]
*SLC7A11-AS1*	chr4: 138,089,014–138,178,177	Negative regulator		Promotes resistance to gemcitabine by repressing SCFβ-TRCP-mediated degradation of NRF2	PDAC cells	[70]
*KRAL*	19q10.14	Positive regulator		Increases sensitivity to fluorouracil by targeting miR-141/KEAP1 axis as ceRNA	HCC SMMC-7721 and HepG2 cells	[71]
*NRAL*	19q10.14	Negative regulator		Enhances resistance to cisplatin for NRF2 by binding to miR-340–5p	HCC SMMC-7721 and HepG2 cells	[72]
*TUG1*	chr22: 30,970,182–30,978,847	Positive regulator		Promotes cisplatin resistance by regulating and stabilizing the NRF2 protein	human EC TE-1 cells	[73]
*MT1DP*	chr16: 56,643,705–56,644,786	Negative regulator		Enhances the sensitivity of NRF2 overexpression to erastin-induced ferroptosis by stabilizing miR-365a-3p	HCC HepG2 cells; NSCLC A549 and H1299 cells	[74,75]

(**A**) * UCSC Genome Browser on Human December 2013 (GRCh38/hg38). Abbreviations: Chr, chromosomal; *SCAL1* or *LUCAT1*, smoke- and cancer-associated lncRNA 1; *NLUCAT1*, nuclear smoke- and cancer-associated lncRNA 1; *NMRAL2P*, NmrA-like redox sensor 2 pseudogene; *TUG1*, Taurine-upregulated gene 1; *LINC00942*, Long Intergenic Nonprotein Coding RNA 942; NRF2, Nuclear factor erythroid 2-related factor 2; ROS, reactive oxygen species; HBE, human bronchial epithelial; ADC, adenocarcinoma; SqCC, squamous cell carcinoma; NQO1, NAD(P)H Quinone Dehydrogenase 1; EMT, epithelial–mesenchymal transition; UCB, urothelial carcinoma of the bladder; TCGA, The Cancer Genome Atlas. (**B**) * UCSC Genome Browser on Human December 2013 (GRCh38/hg38). Abbreviations: *HOTAIR*, Hox transcript antisense intergenic RNA; *MALAT1*, Metastasis-associated lung adenocarcinoma transcript 1; *UCA1*, Urothelial carcinoma-associated 1; *MIAT*, Myocardial infarction-associated transcript 1; *SLC7A11-AS1*, Solute Carrier Family 7 Member 11-antisense 1; *NRAL*, NRF2 regulation-associated lncRNA; *KRAL*, KEAP1 regulation-associated lncRNA; *MT1DP*, metallothionein 1D, pseudogene; MRP2/4, Multidrug resistance-associated protein 2/4; GC-1 spg, germ cell-1 spermatogonial; HUVEC, human umbilical vein endothelial cells; KEAP1, Kelch-Like ECH-Associated Protein 1; RCC, Renal cell carcinoma; ADC, adenocarcinoma; HEK-293, Human embryonic kidney-293; HO-1, Heme oxygenase-1; NF-κB, Nuclear factor-κB; HK-2, Human Kidney-2; VSMC, vascular smooth muscle cells; SCFβ-TRCP, Skp1-cullin-F-box protein-transducin repeat- containing proteins; PDAC, Pancreatic ductal adenocarcinoma cells; ceRNA, competing endogenous RNA; HCC, Hepatocellular carcinoma; EC, esophageal carcinoma; NSCLC, Non small cell lung cancer.

**Table 2:** List of miRNA-positive regulators of NRF2 signaling.

miRNA ID	Direct or Indirect Interaction/ Targets	NRF2-Related Effects	Cell Cultures/Validation Models	Refs.
Let 7b/c	Indirect; *BACH1*, *HO-1*	Participates in negative regulation by suppressing BACH1 and thus stimulating the overexpression of HO-1 via NRF2	HaCaT human keratinocytes cells; HCC (Huh-7 and HepG2) cells	[86,87]
MiR-19b	Indirect; *SIRT-1*	Stimulates the activation of SIRT1 by activating NRF2 cascade and its downstream target genes; enhancing drug hepatotoxicity	Human liver LO2 cells	[88]
MiR-32	Indirect; *PI3K*	Exerts an oncogenic role by inducing the upregulation of NRF2and promoting cells survival	Human RPE cells	[89]
MiR-125b	Indirect; *PRXL2A*	Promotes PRXL2A activation by positive feedback loops involving NRF2 signaling pathway	OSCC (SAS, OECM1, HSC3, FaDu, OC3) cells, HEK-293 and primary NOK cells	[90]
MiR-144	Direct	Regulates the ROS scavenging via NRF2 and stimulates the GSH synthesis	Neuroblastoma SH-SY5Y cells	[91]
MiR-153	Direct	Acts as an oncogene by promoting cell migration and invasion via NRF2 signaling	CRC cells; OSCC (CAL 27) cells and tissues	[92]
MiR-155	Direct	Upregulates NRF2 and its downstream targets (HO-1 and NQO1) by inhibiting apoptosis and mediating ATO resistance	Human lung ADC A549 cells	[93]
MiR-181c	Direct	Enhances the activation of NRF2 and other multiple pro-survival pathways	Human CRC (HT29) cells	[108]
MiR-200a	Indirect; *KEAP1*	Stimulates NRF2 signaling pathway by suppressing KEAP1 and decreasing ROS concentration	OB-6 human osteoblastic cells	[94]
MiR-200c	Indirect; *PI3K/AKT*	Its downregulation provides a feedback mechanism by which NRF2 indirectly regulates E-cadherin and metastasis via PI3K/AKT activation	RPE (ARPE-19) cells	[89]
MiR-365/193b/29b	Direct	Enforces NRF2 and its downstream targets activation	Lymphoblast cells	[96]
MiR-432	Indirect; *KEAP1*	Targets KEAP1 and decreases the sensitivity to cisplatin (CDDP) drug via activation and stabilization of NRF2	ESCC (KYSE170, KYSE770, and KYSE2270) cells	[100]
MiR-455	Indirect; *HDAC2*	Promotes NRF2 expression via its upstream mediator, HDAC2, by ensuring more protection against oxidative injury and activating ARE-related genes (NQO1, HO-1 and GCLC)	MC3T3-E1 and hFOB1.19 osteoblastic cells	[101,102]
MiR-601	Indirect; *CUL3/KEAP1*	Targets CUL3 and concurs into a dependent mechanism of CUL3-KEAP1-NRF2 activation against oxidative damage	RPE (ARPE-19) cells	[103]
MiR-617/592/1207/550	Direct	Exert a pro-oncogenic actions on the NRF2 upregulation; promote cell growth and proliferation by activating antioxidant defense system	ESCC cells; colorectal and liver carcinoma cells; prostate cancer cells	[41,104,105,106,107]

Abbreviations: *BACH1*, BTB Domain And CNC Homolog 1; *HO-1*, Heme oxygenase 1; NRF2, Nuclear factor erythroid 2-related factor 2; HCC, Hepatocellular carcinoma; *SIRT-1*, Sirtuin 1; *PI3K*, Phosphoinositide 3-kinase; RPE, retinal pigment epithelium; *PRXL2A*, Peroxiredoxin-Like 2A; OSCC, Oral squamous cell carcinoma; HEK-293, human embryonic kidney-293; NOK, Normal oral keratinocyte; GSH, Glutathione; CRC, Colorectal cancer; NQO1, NAD(P)H Quinone Dehydrogenase 1; ATO, arsenic trioxide; ADC, Adenocarcinoma; *KEAP1*, Kelch-Like ECH-Associated Protein 1; *AKT*, Protein kinase B or PKB; ESCC, Esophageal squamous cell carcinoma; *HDAC2*, Histone deacetylase 2; ARE, Antioxidant responsive element; GCLC, Glutamate-Cysteine Ligase Catalytic Subunit; *CUL3*, Cullin 3.

**Table 3:** List of miRNA-negative regulators of NRF2 signaling.

miRNA ID	Direct or Indirect Interaction/Targets	NRF2-Related Effects	Cell Cultures/Validation Models	Refs.
MiR-28	Indirect; *KEAP1*	Exerts a negative effect by repressing NRF2 and inhibiting tumor cell growth independently from KEAP1, without any changes at protein level	Human breast MCF-7 and HEK-293 cells	[109]
MiR-92a	Indirect; *KEAP1/ARE*	Its inhibition leads to cell proliferation by decreasing apoptosis-inducing factors and KEAP1 expression with a consequent upregulation of NRF2 and its target genes	HUVECs	[110]
MiR-93	Direct	Acts as a oncogenic regulator by downregulating NRF2 gene and by targeting specific 3′UTR sites with a strong impact on decreasing apoptosis, increasing colony formation and cell migration	Human breast epithelial MCF-10A and neoplastic T47D cells	[111]
MiR-101	Direct	Affects the binding to 3′UTR of NRF2, thus promoting NRF2 overexpression and inhibiting cell proliferation	Human breast MCF-7 cells	[112]
MiR-140	Direct	Targets NRF2 gene by ensuring more radioprotection against TGF-β1-mediated inflammation and fibroblasts differentiation	Human normal lung fibroblast (CCD-19Lu) and mammary epithelial MCF10A cells	[113,114]
MiR-144	Direct	Exerts an oncogenic role by inhibiting NRF2 signaling pathway as well as promotes cell viability, suppresses apoptosis and finally reverses chemoresistance NRF2-related	HL-60 AML and Bel-7402 HCC cells	[115,116]
MiR-148b	Direct	Suppresses cell proliferation and regulates the oxidative stress response by downregulating HIF-1 and NRF2 and inhibiting ERMP1	RL95-2 human endometrial cancer cells	[117]
MiR-153/27a/142/144	Direct	Act as oncogenic miRNAs which decrease apoptosis and successively reinforce cells proliferation via repressing NRF2 and its downstream target genes (GCLC, GSR)	Breast cancer and CRC cell lines; SH-SY5Y neuroblastoma cells	[92,118]
MiR-155	Direct	Accelerates cell malignant transformation by targeting NRF2-mediated oxidative damage and repressing NRF2 and its related gene expressions (GSH, NO and SOD)	16-HBE cells	[119]
MiR-181	Indirect; *SIRT1/PGC-1α*	Suppresses SIRT1/PGC-1α/NRF2 signaling pathway by activating cell apoptosis and oxidative stress	SH-SY5Y neuroblastoma cells	[121]
MiR-340	Direct	Inhibits NRF2-dependent antioxidant pathway and enhancing cells sensitivity to cisplatin	HCC SMMC-7721 and HepG2 cells	[122]
MiR-365	Indirect; *MT1DP*	Represses NRF2 activity via direct binding to its 3′UTR and induce an aggravation of oxidative stress by activating MT1DP	HCC HepG2 cells	[74]
MiR-507/634/450a/129	Direct	Negatively affect NRF2 oncogenic regulation by directly targeting NRF2, thus increasing cisplatin sensitivity and suppressing cell growth	A549 ADC cells	[123,124]

Abbreviations: *KEAP1*, Kelch-Like ECH-Associated Protein 1; NRF2, Nuclear factor erythroid 2-related factor 2; HEK-293, human embryonic kidney-293; *ARE*, Antioxidant responsive element; HUVECs, human umbilical vein endothelial cells; 3′-UTR, 3′-untranslated regions; TGF-β1, Transforming growth factor beta 1; HL-60, human leukemic-60; HCC, hepatocellular cancer; HIF-1, Hypoxia-inducible factor 1; ERMP1, Endoplasmic Reticulum Metallopeptidase 1; GCLC, Glutamate-Cysteine Ligase Catalytic Subunit; GSR, Glutathione reductase; GSH, Glutathione; NO, Nitric oxide; SOD, Superoxide dismutase; HBE, human bronchial epithelial; SIRT1, Sirtuin 1; *PGC-1α*, Peroxisome proliferator-activated receptor gamma coactivator 1-alpha; *MT1DP*, Metallothionein 1D, pseudogene; ADC, Adenocarcinoma.
